# Identification of publicly available data sources to inform the conduct of Health Technology Assessment in India

**DOI:** 10.12688/f1000research.14041.2

**Published:** 2018-04-18

**Authors:** Laura Downey, Neethi Rao, Lorna Guinness, Miqdad Asaria, Shankar Prinja, Anju Sinha, Rajni Kant, Arvind Pandey, Francoise Cluzeau, Kalipso Chalkidou

**Affiliations:** 1 Institute of Global Health Innovation, Imperial College London, London, W2 1NY, UK; 2 International Decision Support Initative, London, W2 1NY, UK; 3 Post Graduate Institute of Medical Education and Research, Chandigarh, 160012, India; 4 Indian Council of Medical Research, New Delhi, 110029, India; 5 National Institute of Medical Statistics , New Delhi, 110058, India; 6 Centre for Global Development , London, SW1Y 4TE, UK

**Keywords:** Health Economics, Health Technology Assessment, India, data

## Abstract

**Background:** Health technology assessment (HTA) provides a globally-accepted and structured approach to synthesising evidence for cost and clinical effectiveness alongside ethical and equity considerations to inform evidence-based priorities. India is one of the most recent countries to formally commit to institutionalising HTA as an integral component of the heath resource allocation decision-making process. The effective conduct of HTA depends on the availability of reliable data.

**Methods**: We draw from our experience of collecting, synthesizing, and analysing health-related datasets in India and internationally, to highlight the complex requirements for undertaking HTA, and explore the availability of such data in India. We first outlined each of the core data components required for the conduct of HTA, and their availability in India, drawing attention to where data can be accessed, and different ways in which researchers can overcome the challenges of missing or low quality data.

**Results**: We grouped data into the following categories: clinical efficacy; cost; epidemiology; quality of life; service use/consumption; and equity. We identified numerous large local data sources containing epidemiological information. There was a marked absence of other locally-collected data necessary for informing HTA, particularly data relating to cost, service use, and quality of life.

**Conclusions: **The introduction of HTA into the health policy space in India provides an opportunity to comprehensively assess the availability and quality of health data capture across the country. While epidemiological information is routinely collected across India, other data inputs necessary for HTA are not readily available. This poses a significant bottleneck to the efficient generation and deployment of HTA into the health decision space. Overcoming these data gaps by strengthening the routine collection of comprehensive and verifiable health data will have important implications not only for embedding economic analyses into the priority setting process, but for strengthening the health system as a whole.

As populations expand and age, increasing demands are made on health resources. Policy makers are required to set priorities and engage in trade-offs to ensure that every dollar, rupee, and rand is spent wisely to maximise value ^1^. This is true for high and low income countries alike, where resources are finite, and all demands upon those finite resources cannot be met. However, in low and middle income countries (LMIC), where some of the largest populations reside, and resources are particularly scarce, making the right trade-offs is arguably of greater importance, where the opportunity cost of making the wrong decision can be a matter of life and death ^1– 3^.

Health Technology Assessment (HTA) provides a globally-accepted and structured approach to synthesising evidence for cost and clinical effectiveness alongside ethical and equity considerations to inform evidence-based priorities ^1, 4^. Almost all high income countries across the world use HTA as a means to systematically consider evidence for clinical and cost effectiveness of new technologies to inform health spending decisions ^5^. Increasingly, systems for generating and utilizing HTA evidence to improve allocative efficiency are also being used in upper middle income countries, such as Thailand ^6, 7^, and South Africa ^8^. Indeed, the use of HTA is credited as one of the key contributors to Thailand’s successful Universal Health Coverage (UHC) program, where the Health Intervention Technology Assessment Program (HITAP) routinely conducts HTA as part of the decision-making process for inclusions and exclusions into the country’s UHC Scheme, and National List of Essential Medicines ^7, 9, 10^. The World Health Organisation signed a health intervention technology assessment resolution in 2014 to support the importance of HTA as an essential component of achieving Universal Health Coverage ^4, 11^, elevating the global recognition of HTA as an important lever for health system reform.

India is one of the most recent countries to formally commit to institutionalising HTA as an integral component of the heath resource allocation decision-making process. This has been recognised in official government policy, including the 12 ^th^ Five Year Plan ^12^, the Niti Aayog 3 year Action Plan ^13^, and the recently released National Health Policy ^14^, marking an important shift in the government’s commitment toward more effective resource allocation for health. The government is in the process of establishing a national HTA body within the Department of Health Research (DHR), a medical research department within the Ministry of Health and Family Welfare, which will use HTA as a means to evaluate the cost effectiveness of new and existing health technologies to support resource allocation decisions at both the central and state levels ^15^.

The effective conduct of HTA depends on the availability of reliable data sources to enable the comparative assessment of the clinical and cost effectiveness of a given intervention within the Indian healthcare context ^16, 17^. Locally-generated data is essential to provide context-specific inputs into a HTA, and the absence of such data poses a serious challenge in building accurate economic models that are statistically robust and truly representative of the context in which they were designed to model and inform. There is a marked absence of strong health information systems in LMIC, and this poses a significant bottleneck to the efficient generation and deployment of HTA into the health decision space ^4, 18^. India is no exception in this respect, where there is limited availability of high-quality clinical, cost, equity, and health related quality of life studies conducted in the country. Overcoming these data challenges will present a significant hurdle in India’s journey towards institutionalising HTA and effectively embedding economic analysis into the priority setting process.

In this paper, we aim to document the currently available local data to meet the needs of undertaking economic analysis in India. We will outline each of the necessary data components required for the conduct of HTA, the availability of such data in India, where data can be accessed, and the different ways in which researchers can overcome the challenges of missing or low quality data. We note that many additional components within the complex HTA ecosystem are of equal importance, including human resources with technical capacity to undertake analyses, and strong political commitment to use HTA evidence to inform policymaking, however we will focus here on data requirements only.

## Methods

**Figure 1.**
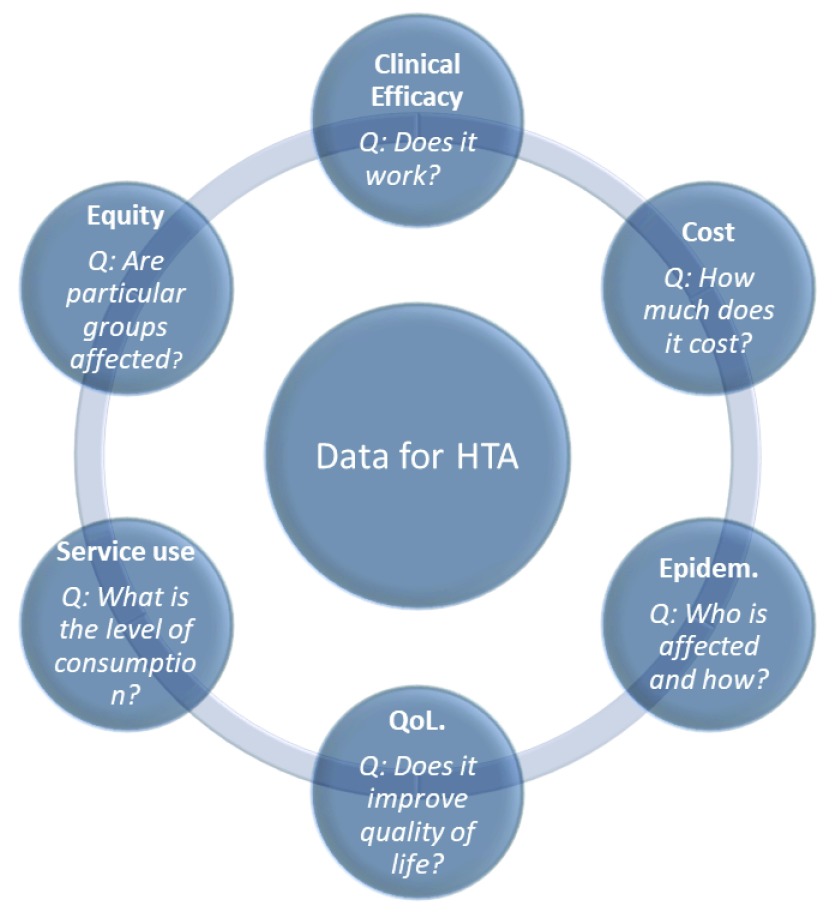
Data requirements for HTA, and key questions that each data component contributes to answering as part of a given economic analyses. Key: QOL = quality of life; Epidem. = epidemiology.

The information presented here is drawn from the experience of the authors in conducting and contributing to health economic analyses, including HTA, in the Indian context. We first identified the key areas of information needed for these types of analyses (see Figure 1). We then used our own experience to document data sources in each of these areas. The authors also approached key informants working within the broad field of evidence science in India for additional inputs and to ensure that the information presented here was sufficiently comprehensive. Finally, each source of data was assessed for its ability to fulfil the needs of HTA. Although, this is not intended to provide an exhaustive account of all databases available across India, this should offer a starting point for researchers wishing to engage in HTA using data from India.

## Results

### Data needs

To construct an economic model for HTA that includes both the costs and impact of a health care programme or intervention within a particular context, data in a range of key areas is needed. We have grouped data needs from these areas into the following categories: clinical efficacy; cost; epidemiology; quality of life; service use/consumption; and equity.

Information on clinical efficacy is necessary in order to understand whether a given intervention is doing what it was intended to do, and how well it achieves this, compared to all reasonable comparators. This is primarily established through randomised controlled trials (RCTs).

In the area of costs, data is needed on the cost of providing a service, or set of services, to providers and patients in order to conduct a comprehensive economic evaluation to determine cost effectiveness. Cost data is outlined in this paper from a health provider and healthcare perspective ^16, 19^, as recommended by the reference case for economic evaluation by the international decision support initiative (iDSI; ^20^). This covers the direct patient and family costs related to the purchase of healthcare, as well as the costs borne by healthcare providers.

Country-specific epidemiological data is what allows researchers to contextualise an economic model and ensure that any conclusions drawn from the results of the model are appropriate for that given context. Epidemiological information includes demographics, vital statistics and burden of disease data. Demographics refers to quantifiable characteristics of a given population, such as gender, geographic location, or socio-economic income status. Vital statistics are information relating to births and deaths of a population, including age at death and the cause. Burden of disease refers to the impact of a health problem on a population, and can be measured by financial cost, mortality, morbidity, or other indicators such as quality adjusted life years (QALYs) or disability adjusted life years (DALYs). Together, this data underpins a large part of what constitutes an economic evaluation model.

Service use data provides information regarding how many people are using a particular service or seeking care which requires a certain intervention. This estimated uptake data is used alongside epidemiological data regarding disease prevalence in economic models to estimate the population impacted by an intervention or service. It is also used in estimating the costs of a particular intervention or health care activity and to inform estimates of the likely budget impact of introducing a new intervention into the health system.

Health Related quality of life data is used to calculate how a given intervention affects quality of life (QoL), and calculate associated quality adjusted life years (QALYs). QALYs are a metric used to estimate an incremental cost effectiveness ratio (ICER), and are the most commonly used metric to represent the impact a therapy has on the length of life while also taking into account any changes in the health-related QoL. QoL datasets are comprised of two separable components – a comprehensive set of health state information based on stated preferences to each QoL dimension, and a weighted utility value generated for that given health state ^21^. The latter component is used to calculate QALYs. It is also possible to utilise burden of disease data, in the form of disability adjusted life years (DALYs), as an alternative to the QALY to capture the disability associated with living with a given condition, and the alleviation of disability after an intervention, in an economic analysis.

Data regarding equitable and equal access, and utilisation of services is essential to allow for ethical information to be considered in the decision-making process alongside evidence of cost effectiveness. In the Indian context, this may relate to inequity as a consequence of gender, caste, religious beliefs, age, geographic location, socio-economic status ^22– 24^.

Further information for each data source is provided below, and summarised in Table 1.

**Table 1.** Summary of key National data sources relevant to the conduct of HTA in India.

HTA-related information	Data Source	Commissioning body	Data Collection method	Equity-relevant information	Website
Epidemiology (communicable disease)	Integrated disease surveillance program	MHFW	Reporting by health and medical officers	Geographic location	http://idsp.nic.in/
Epidemiology, Service use, health expenditure	Health Management Information System	MHFW	Reporting by facility-level data manager	Gender, Geographic location,	https://nrhm-mis.nic.in/SitePages/Home.aspx
Epidemiology, Service Use, OOP spending (for institutional delivery only)	National Family Health Survey (NFHS) 2015/2016,	MHFW	Survey Agency staff trained by IIPS to undertake field work	Location, gender, ethnicity, age, marital status, contraception use, HIV status, health insurance, water/ sanitation access, literacy, female parity	https://dhsprogram.com/data/dataset/India_Standard- DHS_2015.cfm?flag=1
Epidemiology	Sample Registration System (SRS) Census (2011)	MHA	Household census data collected by trained census officers	Location, gender, religion, education, occupation, caste/tribe, language, socio- economic status	http://censusindia.gov.in/Vital_Statistics/SRS/Sample_ Registration_System.aspx
Health and service use/utilization for RMNCH indicators	District Level Household Survey (2011/2012)	MHFW	Regional Agency staff trained by IIPS to undertake field work	Accessibility of services to women and children in rural villages	http://rchiips.org/
Epidemiology, service use, OOP spending (‘Indicators of social consumption: NSSO 2014’)	National Sample Survey Office Census (NSSO; 2014)	MS	Data officers for 6 Zonal Offices, 49 Regional Offices and 118 Sub- Regional Offices collect primary data	Location, Socio economic status, Gender, Rural/urban, Age	http://mospi.nic.in/national-sample-survey-office-nsso
Safety, efficacy, clinical comparator(s)	ICMR Clinical Trials Registry	ICMR	Any medical researcher involved in the conduct of a clinical trial	No	http://ctri.nic.in/Clinicaltrials/advancesearchmain.php
Epidemiology - Cancer	ICMR Cancer Registry Program	ICMR	31 population-based and 29 hospital-based registries	Location, gender, Rural/ urban, Age	http://ncdirindia.org/cancersamiksha/
Health expenditure	National heath Accounts (2014/2015)	MHFW	Secondary data synthesized from many primary sources, full list detailed in document	Public and private sector expenditure	https://mohfw.gov.in/sites/default/files/8949831122147 1416058.pdf
Billing/Price	Database of Indian Health Benefit Packages	WHO India Country Office	Database listing service packages and rates across 22 GFHIS	No	https://public.tableau.com/profile/who.insurance.ben. pack#!/vizhome/BenPackBoxPlot_0/Dashboard2
Billing/Price	Central Government Insurance Scheme Rates information	CGHS	List of reimbursement rates for all procedures covered under CGHS	No	http://msotransparent.nic.in/writereaddata/ mainlinkfile/File811.pdf
Billing/Price	RSBY package reimbursement rates	RSBY	List of reimbursement rates for all procedures covered under RSBY	No	http://www.rsby.gov.in/Documents.aspx?id=4
Equity	Socio-economic and Caste Census (2011)	MRD	Data collected by Government servant enumerators, entered by Central Public Sector Enterprise officer	Socio economic status, caste, religion, living conditions, source of income	http://secc.gov.in/welcome
Epidemiology, Equity	Health States, Progressive India – Health Index report	Niti Aayog	State rankings by index measure derived from various sources	Geography, gender, sex ratio, poverty	http://social.niti.gov.in/hlt-ranking

**Table 1 key**: GFHS, Government Financed Health Insurance Schemes; HTA, Health Technology Assessment; ICMR, Indian Council of Medical Research; MHFW; Ministry of health and family welfare; MRD, ministry of rural development; MS, ministry of statistics;; MHA, Ministry of Home Affairs; OOP, out of pocket spending; SRS, sample registration system; WHO, World Health Organisation.

### Data quality

No formal data quality assessment was carried out by the present authors. However, the Niti Aayog, in collaboration with the Ministry of Health and Family Welfare and the World Bank, have recently published a comprehensive India health index report ^25^, which draws from many of the same data sources outlined below, including the National Family Health Survey and the Health Management Information System portal data, where a thorough quality assessment was undertaken. The authors cite poor data quality as a major limitation of their health index estimates, highlighting a need for urgent improvement in the capture of health and demographic data and the adoption of robust data quality mechanisms across India. As such, all researchers undertaking economic analyses in India should interpret secondary data with caution, and triangulate multiple sources where possible to better understand the data reliability.

### Clinical efficacy and safety

Robust HTA depends on reproducible and verifiable insights into the clinical effectiveness of a given health intervention ^17, 19^. Indeed, it is impossible to establish whether a technology is cost effective if clinical efficacy is unknown or ill-established. Clinical efficacy data is generally available in the published literature for interventions that are presently available in the market. Large RCTs are the optimal source of clinical efficacy data, and these should inform clinical outcome estimates wherever possible. Systematic reviews and Meta analyses of RCTs are the gold standard for deriving clinical outcome and safety metrics. In the case of novel technologies, outcome data may be provided by manufacturers and industry and should be interpreted with caution. The Central Drugs Standard Control Organization (CDSCO) is the National Regulatory Authority in India and publishes a list of approved pharmacological interventions and their indications in India, which is updated regularly. All clinical trials in India are registered in the Clinical Trials Registry of India (CTRI), which is a primary register connected to the International Clinical Trials Registry Platform (ICTRP), an international registry run by the World Health Organization, which houses global data on clinical trials ^26^. The Indian Council of Medical Research (ICMR) is the apex body responsible for the formulation, coordination and promotion of all biomedical research in India, and is a signatory to the WHO joint statement on clinical trials, which mandates that all clinical trials that they fund, co-fund or support would provide public disclosure of results within a year of trial completion ^27^.

The Cochrane Library is the largest international repository of systematic reviews and meta-analyses and should be searched as a first port of call for any pooled statistics regarding clinical efficacy. The ICMR has established an Advanced Centre for evidence-based medicine that hosts the South Asian Cochrane Network and Centre at the Christian Medical College, Vellore, and has procured a national subscription to The Cochrane Library to ensure that it is accessible to scientists across India ^28^. Researchers should conduct a thorough quality appraisal for all evidence used in an economic analyses, using checklists such as CASP or GRADE. The Cochrane Collaboration provides a useful pathway and tutorial for analysts to use to guide approaching grading the quality of evidence. Quality of the data inputs should be transparently reported and taken into consideration when using the evidence from the evaluation to inform decision making.

There may be circumstances where clinical efficacy data should be locally sourced, such as for certain technical surgical interventions, diagnostics that require skilled interpretation, and public health programs that are highly context-specific. Outcomes of these interventions may vary based on training and knowledge of practitioners, and availability of adequate infrastructure and equipment. Researchers should seek expert clinical advice regarding sources of data and appropriateness of transferring international data for use in economic evaluations to inform decision making in India.

## Epidemiological data

### Demographics

The government of India collects epidemiological information periodically through the Health Management Information System (HMIS) and other varied survey sources such as National Family Health Survey (NFHS), District Level Household Survey (DLHS; for reproductive and child health information), and Sample Registration System (SRS) census data. The Most recent round of the NFHS, also known as the District House Survey, data (2015–2016) is the most comprehensive source of primary demographic data in India, where information was collected from over 600,000 households. The Niti Aayog, in collaboration with the Ministry of Health and Family Welfare and the World Bank, have recently published a comprehensive India health index report ^25^, which draws on each of the aforementioned data sources to compile a set of health index statistics for each state and union territory across India. This report highlights key information such as neonatal mortality rate, under-five mortality rate, full immunization coverage, institutional deliveries, and rates of both communicable (TB and HIV) and non-communicable diseases across each State and Union territory.

### Burden of disease

Burden of disease refers to the impact of a health problem on a given population. An important new source of disease burden data for India has recently been published by the Indian Council of Medical Research (ICMR) and the Public health Foundation of India (PHFI), in collaboration with the Institute for Health Metrics and Evaluation (IHME ^29^). This study collected, collated, and synthesized census information, vital registration statistics, Sample Registration System information, large scale national household surveys, cohort studies, disease surveillance data, disease-programme-level data, and administrative records of health services and disease registries in order to estimate disease burden in every state and union territory of India. This marks an important step forward in terms of data availability at the state-level for burden of disease and will provide an invaluable resource for researchers undertaking economic analyses in India.

Further disease-specific epidemiological data is generally available for most conditions in the published literature, though availability varies according to condition. Much of the epidemiological data regarding infectious and communicable disease in India is captured by the integrated disease surveillance program, a Ministry of health and family welfare department under the directorate general of health services. There are also a number of condition-specific programs within the Ministry of Health and family welfare which collect condition-specific information, such as the National program for the control of blindness, or National Programme for Prevention and Control of Cancer, Diabetes, Cardiovascular diseases and Stroke (NPCDCS). The ICMR also runs a network of 26 disease specific institutes across India, such as the Antimicrobial Resistance Surveillance Network, or the Rotavirus Surveillance Network, which collate data on clinical, epidemiological, and virological information, which is used to devise evidence-based treatment guidelines to improve care and monitor and evaluate transmission-modifying interventions such as vaccines. Researchers conducting a HTA study in India should consult the Ministry of Health and Family Welfare website, and individual ICMR institute websites (e.g. National institute of Research in Tuberculosis or National Institute of Malaria Research – see the ICMR website for a full list of institutes) to check whether additional epidemiological information is available for their clinical area of interest.

### Vital statistics (mortality)

The Sample Registration System, part of the government census program, is mandated to collect verbal autopsy data in India. However, the completeness and quality of vital registration data for mortality in India has been highlighted as inadequate, where a recent assessment of the national civil registration and vital statistics systems in place found that available data was incomplete and of poor quality, with a medically-certified cause of death certificate recorded in only ~16% of the total death registered ^30^. In order to address this paucity of reliable data, the ICMR will build on its previous efforts to capture cause of death in 2009 ^31^, and undertake a thorough verbal autopsy study in early 2018. The global collaborative Million Deaths Study, which has been commissioned by the Registrar General of India since 2001, has also collected data in 1.3 million homes in more than 7000 randomly selected areas of India, and provides a useful resource for estimation of mortality data in India ^32, 33^.

### Cost

Cost information is a key component for the process of HTA to inform both cost-effectiveness analysis and budget impact analysis. Cost data is not consistently available across each state and level of the health system in India. Primary research has been undertaken in isolated instances to capture costs for a specific condition or level of care within a given district, however such data is limited.

***Healthcare costs: patient expenditure.*** High healthcare costs combined with low social health insurance penetration has resulted in high out-of-pocket (OOP) expenditure for people across the country, where OOP spending makes up over 80% of health spending in India and represents a major contributor to financial impoverishment ^29, 30^. OOP spending therefore represent a critical component of the costs of health care delivery in India. Additional costs such as costs to other sectors and wages forgone are not covered in this paper. Data for OOP spending was collected as part of the most recent National Sample Survey Office (NSSO; 2014), under the ministry of statistics. Cost data captured by the NSSO relates to expenditure on medicine; reluctance to seek medical services due to financial constraints; reliance on borrowings for medical expenditure; coverage by a health insurance program; expenditure on institutional childbirth; expenditure on hospitalisations and non-hospitalised treatment. In the NSSO survey, OOP can be broken down by broad health condition but are not disease specific. A number of primary research studies have also been published which provide additional data on OOP at the district level across numerous states, and these can be used to complement NSSO.

### Healthcare costs: service delivery

Service delivery data covers cost of all activities related to the provision of healthcare; including medical and allied health professional staff costs, and unit costs of equipment and infrastructure utilised during the delivery of care. Cost data is generally unavailable for service and unit indices, and primary data collection is required in most instances in order to obtain this information.

The World Health Organisation India country office has recently undertaken a comprehensive exercise to collate information on the packages of services covered by 22 different Government funded Health Insurance Schemes across the country, and their service rates. This is the first exercise of its kind to bring together information form myriad schemes across the country into a single database. This information can be used to compare coverage of services across different packages, and the rates at which those services are delivered. This database provides both package rates averaged across schemes by state and disaggregated by individual packages. This provides a highly useful starting point for any researcher undertaking a cost effectiveness analysis in understanding coverage and price of specific services, providing that those services are covered by any government funded scheme. However, it must be noted that these estimates are reflective of tendering price and thus cannot be assumed as accurate reflections of true cost.

Insurance claims data also provide an important source of information on the cost of health care in India. Insurance claims databases contain rich data on secondary care (hospital) claims for individual procedures, however, this data is not routinely made publicly available and reflects the tendered price of healthcare, rather than the actual cost of procuring and providing a given service. There are isolated databases of such information, such as reimbursement rates for the Central Government Health Scheme, or rates of services covered under RSBY. Should this kind of data be made more widely available for public access and scrutiny in future, it would provide an insight into the financing of healthcare in both the public and private sectors, which could help inform more reliable economic models.

There is no single repository for all cost data studies in India, and to date the major source of cost data for public sector has been individual cost studies. In light of the general paucity of cost data , particularly in relation to disease-specific cost information and disaggregated estimates for individual units costs in India, a large primary costing study has been undertaken by the Post Graduate Institute of Medical Education and Research (PGIMER) in Chandigarh, Haryana, where provider cost data has been collected from a sample of over 200 health facilities across 6 states, covering all levels of the health system (more information is available online). Once finalised, this data will be made freely available in the form of an online database resource. The aim of this database is primarily to inform the HTA process, and all researchers across India will be able to use its contents to generate unit cost data to inform their analyses.

At the global level, The WHO has also recognised the lack of reliable national cost data, and developed two tools to support national costing studies: WHO Cost effectiveness and Strategic Planning ( WHO CHOICE) and the WHO OneHealth tool. The former provides country level estimates of unit costs for inpatient and outpatient services for the public and private sectors, but is now largely outdated. The latter attempts to equip policymakers and health service planners with a framework for informing scenario analysis, costing, health impact analysis, budgeting and financing of strategies for all major diseases and health system components. However, it requires local level data to inform these scenarios. At the diseases-specific level, the Global Health Cost Consortium (GHCC) provides an additional tool to improve resources to estimate the costs of TB and HIV programs through encouraging greater transparency and standardisation in costs data collection methods. The GHCC has also developed a unit costs study repository which provides a comprehensive database of a vast array of unit costs by country, including India, related to delivery of HIV and TB services.

### Quality of life

Health Related quality of life data is used to calculate how a given intervention affects quality of life. The European Quality of life 5 dimensions (EQ5D) is the most commonly used generic QoL measure, and has become the cornerstone of HTA in many countries. No comprehensive national dataset for QoL has been collected for India, however, a small number of studies have collected condition-specific EQ5D data ^34^. Researchers undertaking HTA in India who wish to generate QALYs will be required to collect condition-specific EQ5D data to inform their analyses. In the absence of a national QoL tariff for India, utility weights (necessary to calculate a QALY) will need to be transferred from an appropriate existing source, for example that of other countries in the region such as Thailand (see the EuroQol webpage for a list of all published country value sets). It is important to note that there are issues with transferring EQ5D datasets across countries, which can be explained by both methodological differences as well as socio-cultural differences between countries, and researchers should keep this in mind when using international tariffs to estimate local QoL data ^35^.

At the global level, the Global Burden of Disease study (GBD) has collected world-wide data to calculate country-specific burden of disease estimates, including for the Indian population. Burden of disease can then be used to calculate DALYs, whereby one DALY equal the sum of number of years of life lost prematurely (YLLs) and a weighted measure of years lived with disability due to disease or injury (YLDs). YLL uses the life expectancy at the time of death, which can be obtained from the Sample Registration System Census database of vital statistics (2011). YLD is determined by the number of years disabled weighted by level of disability caused by a condition or disease, where the disability weights are derived from the GBD data. Many LMIC have taken to using GBD study estimates to inform economic analyses in the absence of locally-generated quality of life data. The limitations of such an approach should be noted, where the DALY does not easily allow for the modelling of different disease states, and the value judgements used to weight DALY estimates are those of international, rather than local, experts.

### Service use/consumption

Service use data is collected in 2 ways: Through census surveys and household questionnaires; and directly through service providers.

The National Family Health Survey (NFHS), and the National Sample Survey Office (NSSO) census surveys are the largest surveys in India, and collect individual responses regarding consumption of both health and non-health services across the country. Special attention was paid to the collection of health-related information in the 2014 NSSO survey at the request of the Ministry of Health and Family Welfare, where the first national-level information was collected on rates of hospitalisation, medical care received as in-patient in medical institutions, the broad disease areas for which such medical care was sought, the extent of use of Government hospitals, and out of pocket expenditure on medicines and other health-related products incurred was also collected. Expenditure incurred on treatment received from public and private sectors was also accounted for.

While healthcare use data is typically collected by insurers, insurance schemes do not routinely publish this data for public access. Several State-level insurance schemes do publish high-level claims data and report figures on service use, for example Aarogysari scheme in Telangana publishes their annual claims data online each year. Third Party Administrators process most insurance-related claims in India, and hold data regarding institutional-level service use, however this data is also not easily available and not routinely released.

Data regarding uptake of services for particular conditions is also collected by government-sponsored vertical programs, such as the National Aids Control Organization (NACO), and the National Program for the Control of Tuberculosis. HTA researchers should consult the Ministry of Health and Family Welfare website, and individual program websites for their topic area of interest to check whether additional service use information is available.

### Equity – provision and access

There are a number of sources of information across the country that provide information on equity, and a robust analyses of equity information to inform a given HTA should take into account multiple data sources in order to provide a comprehensive assessment of this domain. Data may be extrapolated from large survey samples, such as the National Family Health Survey (NFHS), and the National Sample Survey Office (NSSO), and Health Management Information System portal, where data is available which links demographic information such as gender, geographic location, urban or rural dwelling, age, and socio-economic status to health behaviours and experiences. The Socio-economic and Caste Census was also conducted under the ministry of Rural Development in 2011, which provides additional comprehensive information on these two social determinants of health. Data published by government insurance programs at both the national and state level, such as RSBY, may also be a useful source of information regarding equity in the Indian context, where a number of published studies highlight inequitable access to social health insurance ^36, 37^. The recently published Global Burden of Disease India profile (2017 ^29^), and Niti Aayog ‘Health States’ report ^25^ further highlight health inequity between states across the country and could also be used to inform equity analysis for the conduct of future HTA studies.

### Future research – changing data landscape

India lies at a critical juncture, where political discourse regarding Universal Health Coverage has reinvigorated interest in improving the way in which healthcare is financed, purchased, provisioned, monitored, and governed. The institutionalisation of HTA for setting evidence-based priorities, and the associated strengthening of a robust network of routinely collected and verifiable health data will be a vital lever for systemic reforms to transform India’s fragmented healthcare system. The World Health Organisation urges member states to develop and improve the collection of data on health intervention and technology assessment as part of their Health Intervention Technology Assessment Resolution (2014 ^4^). If the government is committed to leveraging HTA as a tool for realising UHC, health information systems and the platforms for collecting health data will need to be strengthened and adequately governed.

Strengthening data collection should begin at the clinical trial phase, where protocols can be put in place to ensure that cost, safety, efficacy, and socio-demographic information are collected from the earliest phases of technology development. In European countries where it is common process for technologies to go through a process of HTA before entering the market, such data is routinely collected by industry bodies in the development and piloting phases ^5^. Improving the capture of data on health technologies in India, where the health technology industry is one of the largest and most powerful globally, would have important ramifications beyond India for the global health innovation market.

As a Nation leading global advances in technology, telecommunications systems, and mobile devices, there is significant opportunity for India to leverage technological innovation to support health system strengthening. The rollout of Aadhar across the country, which provides each citizen with a unique and identifiable digital identity, gives rise to the possibility of linking health related information to individuals, and consolidating such data for macro-level surveillance and real-time quality improvement. The combination of the these unique digital identifiers and the ubiquitous access to technology further afford the opportunity to aggregate data, expertise, and experience from a variety of sources for health-focused problem-solving a learning health system. Working with large datasets enhances the ability to detect and monitor quality improvement, and can thus meet both clinical and business needs simultaneously. This is of particular importance in the Indian context, where the government relies on harnessing the power of the private sector through mutually beneficial public private partnerships to provide access to high quality publicly-funded healthcare.

It must be noted that much of the data described in this paper is derived from and government-sponsored information systems and census surveys, where specific objectives are present and thus the presence of bias cannot be discounted. Discrepancies between administrative data and independent household surveys from around the world suggest official statistics can systematically exaggerate development progress ^38^, and Indian datasets are not immune to such criticisms ^39^. As India transitions away from its reliance on large-scale international donor-funded studies towards locally-sourced datasets, additional measures for safeguarding data quality and protecting against perverse incentives and the political economy of inaccurate indicators will need to be put in place.

The improved accountability inherent in making health data more transparent can have important indirect consequences in measuring and improving quality of service provision, and reducing low value care ^40^. The ratification of the Indian Clinical Establishment Act in 2010 gives leverage to the government and private citizens alike to demand greater accountability of care across both the public and private sectors, and marks an important step forward towards more transparent health information provision. Improving the way health information is captured, reported, and utilised across the country benefits the entire population: the public are empowered to make informed choices regarding their own health, academics are afforded the opportunity to identify bottlenecks and potential levers for quality improvement, and policy makers are equipped to make more robust, defendable, and evidence-based health policies for improving the health of the population.

## Conclusions

This paper provides a novel and comprehensive framework to assess the availability and quality of local health data required for the conduct of Health Technology Assessment in India. While epidemiological information is routinely collected, other key data inputs such as costs, consumption, and quality of life are not readily available. This poses a significant bottleneck to the efficient generation and deployment of HTA evidence into the health decision space in India. Overcoming these data gaps by strengthening the routine collection of comprehensive and verifiable health data will have important implications not only for embedding HTA into the priority setting process, but for strengthening the Indian health system as a whole.

## Data availability

All data underlying the results are available as part of the article and no additional source data are required.
