# Supplementary material for: Identification of publicly available data sources to inform the conduct of Health Technology Assessment in India
Source: F1000Res. 2018 Apr 18;7:245. Originally published 2018 Feb 28. [Version 2] doi: 10.12688/f1000research.14041.2 (PMC5930391; doi:10.12688/f1000research.14041.2)
Supplement: Peer review history

# Peer review: Referee response for version 2

**DOI:** 10.5256/f1000research.15887.r33271

**Published:** 2018-05-09

**Authors:** Rakesh Kumar Srivastava

**Affiliations:** 1 Wadhwani Initiative for Sustainable Healthcare (WISH) Foundation, New Delhi, India

I have read this submission. I believe that I have an appropriate level of expertise to confirm that it is of an acceptable scientific standard.

# Peer review: Referee response for version 2

**DOI:** 10.5256/f1000research.15887.r31327

**Published:** 2018-05-01

**Authors:** Sumit Mazumdar

**Affiliations:** 1 Centre for Health Economics, University of York, York, UK

**General Comments:** 

 In the Summary Table (Table 1), it is advisable to add a separate column indicating the periodicity or the frequency of availability of these different data sources/datasets. This could help informing about for e.g. the variables/indicators for which a longer time-series may be available.

 Also, the ordering of the information in Table 1 appears rather clumsy. The authors could either follow their own framework of the HTA-associated domains in some sequence or adopt an alphabetically arranged order.

 A significant omission in the Table 1 is the data available from the WHO Survey on Global Ageing and Adult Health (WHO-SAGE) which is a longitudinal study collecting data on mostly older (50+) population but also a sub-sample of adults aged 18-49 years in China, Ghana, India, Mexico, Russia and South Africa. Under the Wave 1 conducted in India during 2006-07, about 11000 individuals surveyed. While there are a number of standardized health measures available from the survey (a new round has been completed in 2015, and data expected soon), its most important aspect is the availability of a set of health vignettes, and rare availability of a number of Quality of Life measures such as the Health-related Quality of Life (HRQoL) scale. Details are available here.

 The other useful data source – particularly for the elderly population (aged 60+) – is the UNFPA-supported database under the Building Knowledge Base on Population Ageing in India (BKPAI). This is undoubtedly the richest source of data available for a nationally representative sample of the elderly population, providing rich information on an extensive set of physical, functional and mental health conditions, apart from standard information on sources and costs of medical care.

**Specific Comments: **

1. Epidemiological data: Under ‘Demographics’, it may be useful to mention that the Indian NFHS is based on the USAID-supported Demographic and Health Survey (DHS), currently covering 90 countries. Also while referring to the recent NFHS, it is wrongly referred as ‘District House Survey’ (The District Level Household Survey-DLHS is a distinct entity, most likely being discontinued after the Round 4 conducted in 2012-13). Also, can mention the NCDs covered in the NFHS (anaemia, hypertension, blood glucose levels/hyperglycaemia). 
2. Although it has been mentioned in the summary table, it may be useful to indicate in the discussion on burden of diseases (BOD) that data from the IDSP (and mostly from other disease-specific national programmes) are population averages, or aggregate prevalence/incidence levels. I am not aware of the availability of any large-scale, nationally representative disease-specific survey by ICMR, but the periodic NSSO health surveys are probably India’s only health data from which disaggregated BOD estimates can be derived for different socioeconomic (education, social groups, economic status) and demographic parameters. For rarer diseases, sample sizes can be an issue, but broad patterns should be well discernible.
3. Under the ‘Costs’ it is important to recognize and distinguish between ‘private’ costs or expenditures (under which the OOP expenditure falls) and ‘public’ expenditure. This has been discussed to some extent in the following section on ‘Healthcare costs’ but that is only limited too publicly-funded health insurance schemes. Hence, it is suggested the authors include the sources of aggregate health expenditure for e.g. the National Health Accounts which have already mentioned in Table 1, and other time-series sources such as the Reserve Bank of India compendium of state budget documents, from the health-systems perspective of the costs in the context of HTA studies.
4. In pg 9, under ‘service use’ there has been an erroneous mention of the word ‘census’ after NFHS, NSSO
5. In the discussion part, the authors might consider the suggestion – in the light of the observation they themselves make – to the insurers, including the relevant public/quasi-public agencies to make available suitably anonymized data from public health insurance schemes, given India’s increasing orientation towards such a model. 
6. The authors need to consider – as a matter of style – the use of upper and sentence cases while referring to terms such as state, national, government, ministry etc. a single, common style needs to be followed, and also for acronyms such as AADHAR.

I have read this submission. I believe that I have an appropriate level of expertise to confirm that it is of an acceptable scientific standard.

# Peer review: Referee response for version 1

**DOI:** 10.5256/f1000research.15265.r31324

**Published:** 2018-04-10

**Authors:** Rakesh Kumar Srivastava, Kavita Kachroo

**Affiliations:** 1 Wadhwani Initiative for Sustainable Healthcare (WISH) Foundation, New Delhi, India

The present article is the first research/review article on data availability for HTA in India. It has attempted to capture the gaps in the availability of data – in details, scope, and quality & inter state and national comparability, so that it can be used as base line to initiate HTA for major public health interventions. Since it is not a standard research article, its result have been quoted under the sub-head of need, quality, efficacy, safety, epidemiology, disease burden, cost, quality of life, service use and equity. It also suggest for taking advantage of digital Revolution…..of data generators to strengthening the way in which the information is collected, reported and utilized.  A suggestion for future research is also included in the article.

 The conclusion mentioned “towards commitment of GOI for institutionalizing HTA as integral component of health resource allocation decision”. With this conclusion from the research article, the relevance of details of Data Needs as given on page 4, becomes conclusive and unidirectional when it writes that “the cost data is outlined …..by the international decision support initiative (iDSI)”. Point of improvement are required for areas where suggestions has been given for iDSI which can be seen under the head of data need and service delivery cost. It is suggested to incorporate others if available without specific mention of one.

We have read this submission. We believe that we have an appropriate level of expertise to confirm that it is of an acceptable scientific standard, however we have significant reservations, as outlined above.

# Peer review: Referee response for version 1

**DOI:** 10.5256/f1000research.15265.r31325

**Published:** 2018-03-16

**Authors:** Malaisamy Muniyandi

**Affiliations:** 1 ICMR-National Institute of Research in Tuberculosis(NIRT), Chennai, India

The authors have focussed a very important issue in the context of Health Technology Assessment in India. Data availability and accessibility from both public and private sources is a big challenge while conducting HTA in any discipline. The authors have appropriately described their valid experiences in this article. The information provided in this article will be of use for practitioners of HTA in India.

 I recommend the authors to consider the following issues and address them:

- Authors can consider the programmatic data which is available. For example Revised National Tuberculosis Control Programme had collected valuable patient wise data and programmatic management data including resources/technologies invested and used. Similarly other programmes like National AIDS Control Programme need to be looked into for data sources.
- Data available with non-government organisations which have been working on various health related interventions need to focused on.
- Data from academic institutions like IIT and universities where students and faculties are engaging in innovations and newer technologies need to be explored.

I have read this submission. I believe that I have an appropriate level of expertise to confirm that it is of an acceptable scientific standard.
